# Current status of surgery-first approach (part III): the use of 3D technology and the implication in obstructive sleep apnea

**DOI:** 10.1186/s40902-020-0245-x

**Published:** 2020-01-31

**Authors:** Junho Jung, Seung-Hwan Moon, Yong-Dae Kwon

**Affiliations:** 1 Department of Oral & Maxillofacial Surgery, School of Dentistry, Kyung Hee University, Dongdaemun-gu, Seoul, 02447 South Korea; 2 Department of Oral & Maxillofacial Surgery, Graduate School, Kyung Hee University, Seoul, South Korea

## Abstract

Considering psychosocial needs of patients, it is not surprising that surgery-first approach (SFA) is becoming more popular than ever. Although the concept of SFA was introduced a few decades ago, the limitation of analysis method based on two-dimensional images makes surgeons reluctant to choose SFA. Recently, the advancement of three-dimensional technology allows us to perform SFA even without minimal pre-surgical orthodontic treatment, and the prediction of surgical outcome became more accurate, especially in obstructive sleep apnea (OSA) patients to whom the advantages of SFA should be more significant. Here, we describe the current trend of SFA and its implication in OSA patients.

## Background

Preparation of orthodontics prior to surgery was rare for patients who needed orthognathic surgery until the 1960s. Patients and clinicians want the best esthetic and occlusal results, leading to the most common current treatment approaches for pre-operative orthodontic decompensation and tooth alignment. Disadvantages of performing orthodontic treatment before and after corrective surgery include long treatment times about 7 to 47 months, dental caries, gingival recession, and root resorption. Other complications related with pre-operative orthodontic treatment are temporary facial appearance worsening and masticatory discomfort. After all preparation is complete, if the patient refuses surgery, the results will be devastating. Many new techniques and methods have been introduced since the first orthognathic surgery was performed by Hullihen in 1848. As Kondo and Aoba show, the limits of severe malocclusion are increasing with orthodontic treatment alone, but skeletal imbalances remain. The current concept of “surgery-first and orthodontic treatment second” to reduce the inconveniences and problems of pre-operative orthodontics was proposed a few decades ago, and a recent development of 3D technology allows the accurate surgical planning and the prediction of post-operative tooth movement. Therefore, more and more surgeons and orthodontists are shifting in favor of surgery-first approach (SFA) when it meets the criteria. In this series of reviews, we want to discuss about recent trend and implication in obstructive sleep apnea.

## Review

### Early surgery vs. surgery first

The patient “prototype” for orthognathic surgery has been changed. The desire to improve facial esthetics, rather than just correcting a dysfunctional occlusion has become main the motivation for treatment in many cases. The desire for esthetic improvement, coupled with the general perception of surgery as safe and predictable, broadens the number and age range of patients who become involved in orthodontic or combined orthodontic surgery. In addition, patient does not like pre-operative orthodontic treatment due to the duration of treatment, and patient’s demand for short treatment duration is increasing.

#### Indications

Conventional “early surgery” and “surgery first” both approaches have indications and treatment planning considerations. We should choose the suitable method by knowing the differences. Conventional early surgery is usually selected when indication—(1) minimal crowding in the anterior teeth, (2) favorable curve of spee, and (3) normal range of angle between the basal bone to upper and lower incisors—for “surgery first” was not met, but the patient wants an immediate esthetic change [1]. In this treatment concept, tooth extraction due to facial asymmetry and the presence of severe crowding and complex 3D dental compensations required at least partial orthodontic preparation. Many patients who are orthodontically compensated individuals who are unhappy with the esthetic outcome want a surgical correction of their deformity. Patients need to be informed that the SFA may require more surgical intervention whereas it has faster improvement of the facial profile and accelerates the treatment process. Patients with problems in the temporomandibular joint or periodontal disease may not be a candidate for surgery-first approach [2]. For the mild temporomandibular disorder cases, surgery-first approach with intraoral vertical ramus osteotomy is considered. The disadvantage of the surgery-first approach with intraoral vertical ramus osteotomy is a 4-week intermaxillary fixation [3]. From several clinical studies, type I collagen and serum level of alkaline phosphatase that can be considered as indicators for bone turnover are increased until 3 to 4 months postoperatively [4, 5]. This change is called to regional acceleratory phenomenon (RAP). Since the peak activity of RAP is 1 to 2 months after surgery and lasts until 6 to 24 months after operation [4], 4-week intermaxillary fixation will delay the initiation of post-operative orthodontic treatment. Correcting the mandibular retrognathism with deep occlusion, extraction cases, and narrow palatal arch is very difficult without pre-operative orthodontic treatment [1, 5]. Most patients not recommended for surgery-first approach show complicated post-operative orthodontic treatment.

#### Orthodontic preparation differences

Traditionally, orthodontic surgery has performed orthodontic treatment prior to surgery to eliminate dental compensation caused by skeletal discrepancies and to improve post-operative occlusion stability.

Usually, pre-surgical phase is relatively longer than the post-surgical phase and can cause progressive deterioration of facial esthetics and dental function [6, 7]. Early surgery, in other words, minimal pre-surgical orthodontics (MPO) concept has been proposed to reduce the instability of post-surgical occlusion and efficiently increase the predictability of the surgical results [8, 9]. MPO is performed to minimize occlusal interference during surgery by intruding the extruded posterior teeth, controlling the torque of the posterior teeth, and coordinating the arches for surgery. Although the SFA does not involve pre-operative orthodontics, fixed orthodontic instruments are often placed preoperatively to facilitate post-operative orthodontic treatment. If these are not placed prior to surgery, placement in the immediate post-operative period is often very difficult for the patients, as they may swell during this time, feel discomfort, and have limited ability to open the mouth during this time. For post-operative orthodontics, there are several options for pre-operative preparation for the SFA according to the literature [10, 11] as follows: (1) pre-operative placement of surgical arch bar, without orthodontic arch wire; (2) pre-operative placement of anchor screws, without orthodontic arch wire; (3) pre-operative placement of light round or light rectangular wire (with/without screws or anchor plates); and (4) pre-operative placement of conventional passive, rectangular wires attached with surgical hook (with/without anchor screws).

Additional maxillomandibular fixation (MMF) screws or anchor miniplates are frequently used, as surgical hooks cannot be placed on light round or weak square wires. Passive adaptation of conventional rectangular stainless-steel wires is not easy for patients with severe crowding or spacing. Alternatively, Kobayashi hooks or eyelet wires can be used for intraoperative MMF or post-operative guiding elastics. When surgery is planned, full brackets with surgical wires are strongly preferred to light wires or anchor screws. In contrast to conventional approach, it is convenient to use an anchor screw for intraoperative MMF, and it is possible to place a light round/rectangular wire to initiate the post-operative orthodontics easy and quick [12].

#### Occlusion and management

There are some considerations for occlusion. In early surgery, post-operative occlusion is known to be relatively stable and needs short post-operative orthodontic treatment. Considering the purpose of orthodontic treatment before surgery was to reveal the true extent of skeletal deformation by restoring the correct teeth-bone position. The process of decompensation includes arch adjustment, removal of crowding, and tooth inclination correction [1, 5]. However, complete decompensation may not be possible due to the chewing function and strength, and the direction of the natural compensation process. This occurs in the opposite direction of orthodontic treatment before surgery. This explains why post-operative orthodontic treatment is generally required in addition to pre-operative treatment. In contrast, given the direction of natural compensation after orthodontic surgery, post-operative orthodontic treatment appears to be consistent with the natural process. For example, after orthognathic surgery in a class III patient without decompensation treatment, the mandibular incisors are compensated for the tongue. This is in accordance with dental decompensation procedures. The mandibular incisor will go for labioversion which is also in accordance with the dental decompensation procedures. Therefore, even after pre-operative correction, complete stability of post-operative occlusion cannot be obtained and evaluation of post-operative orthodontic treatment for stable occlusion should be accompanied [13].

In the SFA, because post-operative occlusion is totally dependent on the surgical splint, more attention should be made in post-operative physical therapy or guidance of post-operative occlusion. Also, when occlusal interference occurs before surgery, surgeons often adjust occlusion by eliminating the premature contacts or high points immediately before or during orthognathic surgery [14]. Although these occlusal interferences can be minimized by pre-operative orthodontics, the SFA requires more frequent reduction of occlusal interferences during the surgery in comparison. Improper occlusion after SFA can lead to unexpected mandibular positions in the posture. These can affect the long-term outcomes of the surgery. Wearing and adjusting the surgical splint after surgery is an important step for stable occlusion and long-term skeletal stability. If significant occlusal discrepancy is expected after surgery, buildup of occlusal resin bite blocks to stabilize the immediate post-operative occlusion or modified post-operative wafer should be strongly considered. The limitation of surgery-first approach is mostly associated with occlusion. Without the help of 3D virtual imaging and simulation surgery, complex cases cannot be treated by surgery-first approach [15]. As post-operative occlusion is generally unstable in surgery-first approach, surgical wafer should be maintained for guiding post-operative mandibular movement.

### Application of 3D planning

Orthognathic surgery requires a precise surgical plan for the correction of craniofacial deformity; not to mention, it is more important to surgery-first approach (SFA) performed without pre-surgical orthodontic treatment. Pre-surgical planning for orthognathic surgery using two-dimensional lateral and posteroanterior cephalometric radiographs, dental casts, face bow, and extraoral and intraoral photographs has been considered as a gold standard. There are no doubts that the conventional planning methods provide sound clinical and esthetic results in orthognathic surgery and still widely used in many clinics. However, inherent errors originated from the impression of dentition, the fabrication of dental casts and face bow transfer should exist [16, 17], and two-dimensional cephalometric tracing causes measurement errors [18]. Furthermore, the linear measurements on lateral and posteroanterior cephalograms inevitably have differences on the actual distance, and the prediction of three-dimensional (3D) movement of the maxilla and mandible after orthognathic surgery cannot be accurate as it is planned on the two-dimensional (2D) plane [19–21].

#### 3D virtual planning

The introduction of computed tomography enables 3D modeling of facial and dental structure, and it facilitates the shift of surgical planning technique from 2D to 3D. Recent 3D imaging software even allows combining of facial soft tissues, the underlying skeleton, and dentition together, and it is also applicable and beneficial for the SFA. Although, multi-detector computed tomography (MDCT) and cone-beam computed tomography (CBCT) have disadvantages to scan accurate teeth surface and occlusion relationship due to artifacts and geometrical collision, an intraoral dentition scanning or a surface scan of dental casts combined with a three dimensionally reconstructed skeletal models can dissolve the problems [22, 23].

Several studies have been added up to establish a methodologic process of the 3D virtual planning of orthognathic surgery [19, 22, 24, 25]. It is a stepwise planning including diagnosis, 3D cephalometric measurements, virtual planning and osteotomy, and the prediction of the dentoskeletal movements and soft tissue changes [24, 26–28]. Preoperatively, MDCT or CBCT data were obtained, and a scanned dental cast data was fused to the virtual skeletal model by voxel-based matching methods [22]. After 3D cephalometric analysis was performed, virtual osteotomies including Le Fort I osteotomy, bilateral sagittal split, or vertical ramus osteotomy were simulated using 3D imaging software, e.g., Dolphin 3D Imaging®, Simplant O&O®, providing 3D tools for measurement of hard and soft tissues, osteotomy simulation, and image superimposition. The segments of maxilla and mandible are virtually repositioned to correct skeletal deformity as planned and can be superimposed and compared with the original positions of the segments. The final occlusion can be obtained by either an occlusion positioned exclusively digitally or a scanned dental cast under final occlusal position. Schneider et al. described that the best possible occlusion was achieved with the help of enlarging the 4 K display and a color-coded collision warning, preventing any occlusal interference [28]. Based on the simulated maxilla and mandible position, and their occlusion relationship, the design and fabrication of surgical wafers were performed using computer-aided design and manufacturing software and 3D printers. With the help of CAD/CAM methods, the process can be more time-saving and precise than conventional methods to fabricate surgical wafers [28].

Several studies advocate the accuracy of the 3D planning for orthognathic surgery compared to the conventional 2D methods. Schneider et al. concluded in their prospective randomized trial that there were significant differences in accuracy and in the duration of the operation in favor of the 3D surgical planning [28]. Bengtsson et al. indicated that 2D and 3D planning techniques both showed a high accuracy in predicting facial outcome; however, 3D planning has an obvious advantage in asymmetric patients [29]. Chin et al. reported that no significant deviation between 3D surgical plan and post-operative result was detected [22]. Xia et al. demonstrated 0.9 mm and 1.7° of differences between pre-operative plan and post-operative outcome, which is not clinically significant [30]. Hsu et al. showed a similar result with the largest root mean square deviation (RMSD) of 1.0 mm and 1.5° for the maxilla, and 1.1 mm and 1.8° for the mandible, in their prospective multicenter study [31].

#### 3D surgical planning in SFA

3D surgical planning should be more advantageous in SFA. The SFA can hardly achieve an accurate prediction of post-operative result, since dental occlusion without pre-operative orthodontic treatment cannot be used as a guide to establish surgical plan. Although there was no study directly comparing 2D and 3D planning method in the SFA, Tran et al. reported that a virtual surgical planning and 3D-printed surgical splints offers an accurate result in the SFA [25], and Uribe et al. showed favorable esthetic and occlusal outcomes on the surgical correction of facial asymmetry with the SFA [15]. Im et al. reported a successful case of the SFA using a three-dimensional virtual setup and surgical simulation [32]. However, there should be more evidence with prospective, randomized, and multicenter studies to confirm the benefit of 3D planning in the SFA.

#### Virtual setup

If SFA is planned instead of orthodontic treatment first approach, a dental “setup” is occasionally required to precisely predict the occlusion after post-operative orthodontic treatment. Based on the occlusion simulated by the dental setup, the movement of the mandible is determined in case of the Maxilla first orthognathic surgery. Conventionally, each tooth is separated from a plaster model and repositioned to simulate potential therapeutic objectives within the framework of orthodontic treatment planning [33]. However, it is time-consuming requiring a laboratory procedure and difficult to reproduce the same setup. Recent development of 3D technology has enabled to produce virtual setup [34, 35]. Barreto et al. concluded that the virtual setup is as effective and accurate as the conventional setup and reliably reproduced in actual orthodontic treatment [35]. Camardella et al. also reported its advantages of digital storage, digital communication, reproducibility, time efficiency, and combinability with skeletal digital data [34]. Although it requires considerable time and training to handle digital models, the virtual setup is expected to eventually replace the conventional setup in the near future.

### OSA as an indication of surgery-first approach

OSA is characterized by symptoms that result from the recurrent sleep-associated collapse of the pharyngeal airway and lead to symptoms such as hypoxemia, hypercapnia, and fluctuations in intrathoracic pressure caused by increased respiratory effort, with arousal from sleep required to reestablish airway patency [36, 37]. Multiple physiological processes lead to OSA and the pathology of OSA is complicated. Factors that play an important role in the development of OSA are reduced pharyngeal dilator expansion and upper airway anatomy abnormality. Mandibular advancement was first proposed by West and colleagues in 1979, as an alternative to tracheostomy for the treatment of OSA [38]. In various modalities, maxillomandibular surgery has obtained popularity and known as an effective surgery in the mid-1980s. By the early 1980s, Riley et al. had further documented the value of mandibular advancement surgery as a specifically applied for the treatment of OSA [39, 40]. They proposed simultaneous maxillary and mandibular advancement to gain the upper airway space and to reduce the apnea episodes who suffer with OSA. This surgical approach has been consistently shown to be very effective modality for opening the total upper airway passage for patients with OSA.

**Fig. 1:**
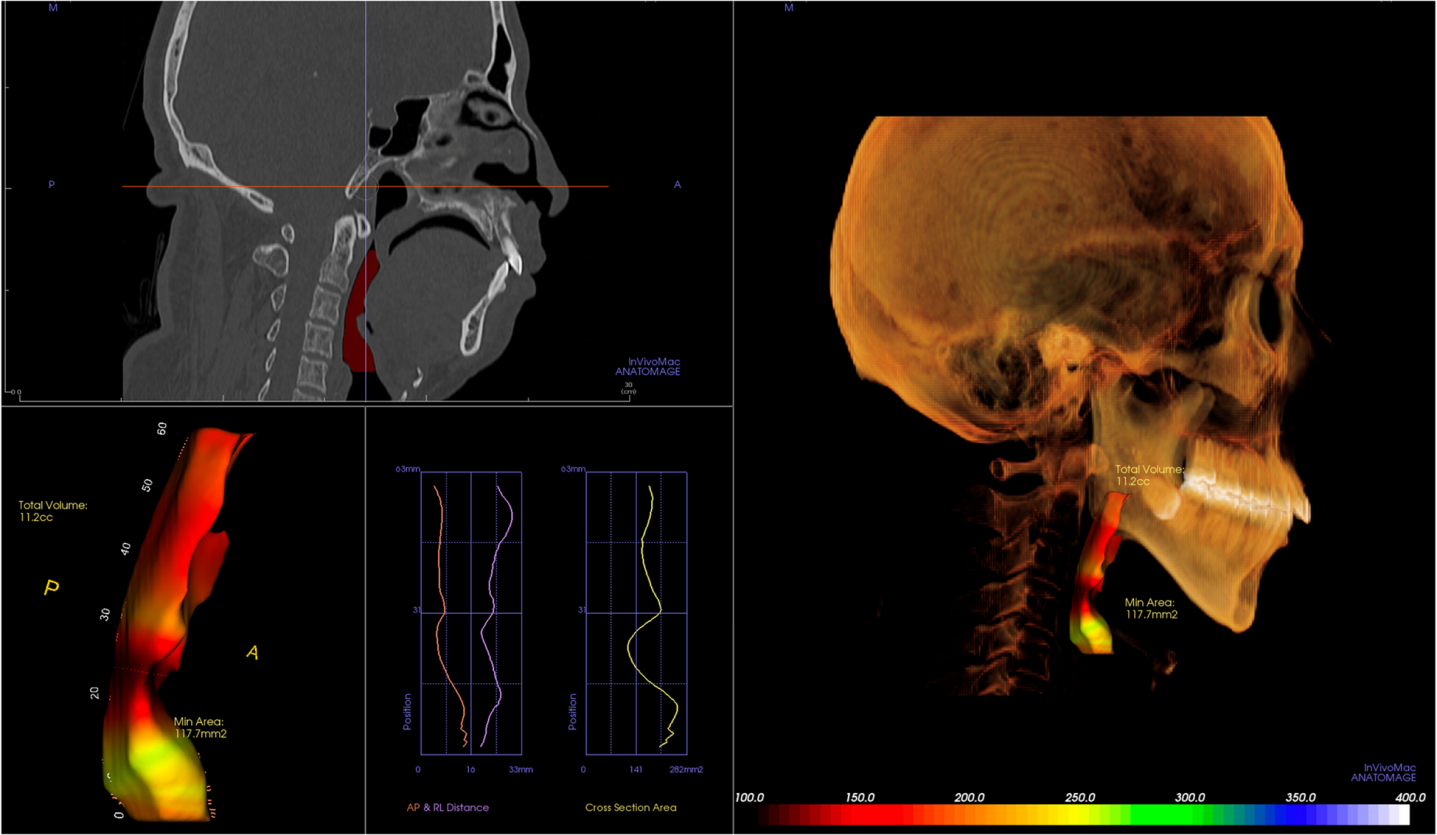
The volume and shape of the airway can be reconstructed from the CT dataset. Minimal cross-sectional area can be identified and this can be a candidate for a target of a surgery

Evaluating OSA patients, airway volume is routinely checked, and reconstructed 3D CT image can provide the pre-operative airway volume (Fig. 1). Using a 3D software, the airway volume and the minimal airway cross-section can be obtained (Fig. 1), and this may help the patient understand his/her upper airway configuration. Often, surgeons can assume where the possible anatomic level of obstruction is located although the 3D images can give only static information.

For severe OSA patients, who needs immediate airway increase, “surgery-first” concepts can be good treatment options. The maxillomandibular surgical procedure includes a standard Le Fort I combined with mandibular sagittal split osteotomy for advancement of the maxilla and mandible together, which will increase the airway space automatically as it moves the base of the tongue and soft palate anterior, thus reducing upper airway resistance [41]. Before the surgery, it is necessary to decide whether the dental occlusion is going to be changed or not. However, for most patients, the sleep apnea and related problems (sleepiness, tiredness, cardiovascular comorbidities) are a priority and the orthodontic treatment can be postponed or minimally done in many cases. Some patients often choose to keep their occlusion status the same. Accurate prediction of surgical outcome is possible using 3D planning and simulation, so surgeons can control a delicate balance between facial esthetic change and resolution of OSA. However, even if patients made decision not to change their dental occlusion, about 5% of patients developed new malocclusion after orthognathic surgery, which needs to be corrected by post-operative orthodontic treatment [42]. In case of OSA, because the primary outcome of the surgery is the reduction of apnea/hypopnea which have adverse impact on systemic health, the post-operative prediction in this context is limited.

Looking back the traditional maxillomandibular advancement (MMA), the osteotomized maxillomandibular complex were simply advanced to gain upper airway patency, but counterclockwise rotation of the maxillomandibular complex which is namely rotational MMA became popular [43–45]. Before the virtual surgery planning (VSP) came out, surgeon’s experience and insight were the most important part to get optimal outcome. For novice surgeons, it is quite difficult to control three-dimensional position of the maxillomandibular complex. The conventional model surgery using an articulator may be lack of consistency because of innate errors coming from manual works.

The location of the center of rotation is one of the important parts when planning a rotational MMA. Depending on the location of this point, the amount of horizontal and vertical movement would differ among the landmarks. Because the conventional paper surgery and model surgery would make errors especially in this rotational movement of MMC, VSP is of great help during setting up the surgery plans.

**Fig. 2:**
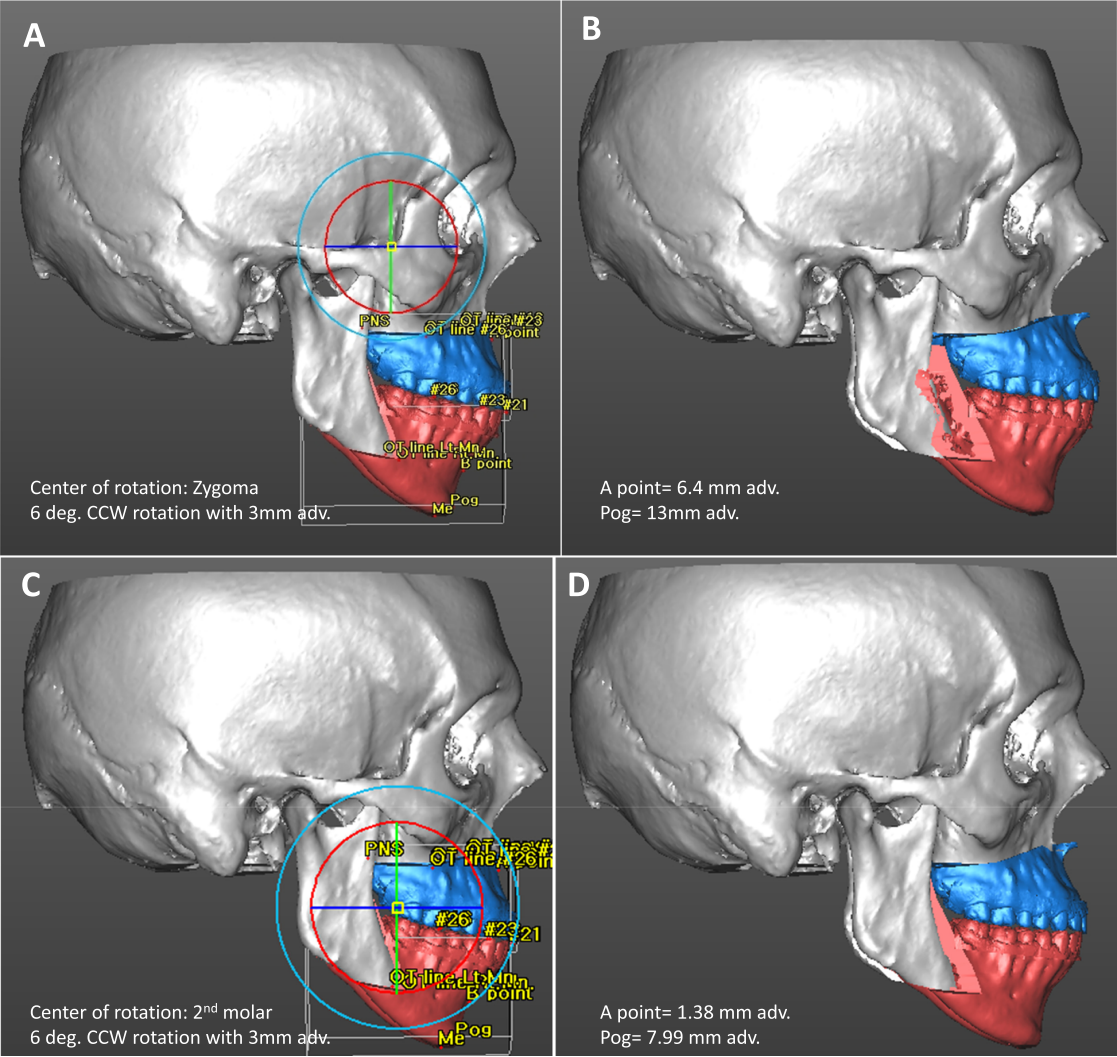
In an OSA patient, minor bite problem can be deferred after correction of the airway problem. According to the location of center of rotation, the amount of advancement can vary. A rotation point located in zygomatic area (**a**) can mobilize Pog more anteriorly (**b**) than those located in distal part of upper 2nd molar (**c**, **d**). In this context, the location of rotation point should be determined under consideration of facial esthetics and airway regaining

Varying the center of rotation during VSP, surgeon can easily compare the facial changes between each planning. In the VSP plan shown in Fig. 2, the amount of advancement is different depending on the rotation centers and we can immediately recognize the change. Therefore, VSP is also beneficial in the treatment planning procedure for OSA patients and it would be easy to find a balance between the airway regaining and facial esthetics through the images from VSP with which we can increase the patients’ understanding and raise treatment acceptance rate.

## Conclusion

Surgery-first approach (SFA) is becoming increasingly popular with the help of 3D virtual planning of orthognathic surgery and dental setup. Although the concept of SFA was introduced in the remote past, precise and correct prediction of post-operative outcome without pre-surgical orthodontic treatment was hard to achieve, before the development of 3D planning and simulation technology. There is no doubt for patients to improve facial skeletal problem earlier than correcting a malocclusion in many cases. The patient-centered approach is now possible without the concerns of unfavorable result. Many clinicians advocate the advantages of SFA, not mentioning the esthetic standpoint. The overall orthodontic treatment period for conventional approach was ranged from 22 months to 36 months [46–48]. On the other hand, the treatment period for SFA was ranged from 10 months to 14 months [48, 49]. In addition, the possibility of overcorrection was suggested as an advantage of SFA [50]. The relapse after orthognathic surgery was commonly mentioned in many studies. However, the overcorrection of mandible is usually not possible in conventional approach since post-surgical occlusion was already determined by pre-surgical treatment, and it was the goal for the conventional approach. In SFA, the occlusion after surgery is not stable, and it allows slight accentuation of the surgical movement of the mandible [50]. Therefore, the slight relapse can be compensated by the overcorrection. In case of an OSA patient, SFA is often recommended. The patient usually has stable occlusion, so the advancement or counterclockwise rotation of maxillomandibular complex is achieved without the change of occlusion relationship. Moreover, the immediate correction of OSA condition is sometimes required to be the first consideration for the patients, and minor orthodontic treatment can be performed afterwards. The prediction of facial esthetic change is possible using 3D planning and simulation. Especially, facial changes often may be an untoward effect, so the prediction provided by VSP would be mandatory for the discussion with the patients. Therefore, the amount of maxillomandibular movement and the possible facial change can be readily consulted with the patient, and the surgeon can seek a balance between the esthetic acceptance and the resolution of OSA. New technology provides accuracy and convenience for surgeons. However, thorough pre-surgical evaluation is still mandatory with orthodontists to avoid unfavorable result and post-surgical complications.

## Data Availability

None

## Abbreviations

2D: Two-dimensional
3D: Three-dimensional
CBCT: Cone-beam computed tomography
MDCT: Multi-detector computed tomography
MMA: Maxillomandibular advancement
MMC: Maxillomandibular complex
MMF: Maxillomandibular fixation
MPO: Minimal pre-surgical orthodontics
OSA: Obstructive sleep apnea
RMSD: Root mean square deviation
SFA: Surgery-first approach
VSP: Virtual surgery planning
